# Resolving the systematics of Richtersiidae by multilocus phylogeny and an integrative redescription of the nominal species for the genus *Crenubiotus* (Tardigrada)

**DOI:** 10.1038/s41598-020-75962-1

**Published:** 2020-11-10

**Authors:** Daniel Stec, Matteo Vecchi, Wojciech Maciejowski, Łukasz Michalczyk

**Affiliations:** 1 Institute of Zoology and Biomedical Research, Jagiellonian University, Gronostajowa 9, 30-387 Kraków, Poland; 2 Department of Biological and Environmental Science, University of Jyvaskyla, PO Box 35, Jyvaskyla, 40014 Finland; 3 Institute of the Middle and Far East, Jagiellonian University, Oleandry 2a, 30-063 Kraków, Poland

**Keywords:** Evolution, Zoology

## Abstract

The family Richtersiidae, although established recently with the use of phylogenetic methods, was considered potentially paraphyletic at the time of its erection. Until now, the family comprised four genera, *Richtersius*, *Diaforobiotus*, *Adorybiotus* and a newly erected genus *Crenubiotus*. However, the genetic characterisation for the latter two genera was very limited or absent. To address concerns about the phylogenetic affinity of these two genera, we present a multilocus phylogeny of the families Richtersiidae and Murrayidae based on four molecular markers (18S rRNA, 28S rRNA, ITS-2 and COI). Our results show a distinct evolutionary lineage composed of *Adorybiotus* and *Crenubiotus*, which is sister to Murrayidae. In order to accommodate the phylogenetic and morphological distinctiveness of this lineage, we erect a new family, Adorybiotidae **fam. nov.** The new taxon differs morphologically from other families in the superfamily Macrobiotoidea by a unique combination of traits: (1) the presence of tubercles/cushions with aggregations of microgranules on their surfaces present on all legs and on the dorso-caudal cuticle, (2) a system of internal septa in claws, and (3) buccal apparatus morphology. Moreover, in order to stabilise the taxonomy and nomenclature in the genus *Crenubiotus*, we redescribe its type species, *Crenubiotus crenulatus*, by means of integrative taxonomy and designate a new neotype based on a population from the original *terra typica*.

## Introduction

Tardigrades are a phylum of microinvertebrates which are found in freshwater, marine and limno-terrestrial environments throughout the world^1^. The first formally described tardigrade was *Macrobiotus hufelandi* C.A.S. Schultze, 1834^2^ and currently over 1300 nominal taxa are recognised within the phylum^3–5^. Although research on tardigrade systematics started almost two centuries ago, only recently have studies aided by molecular phylogenetics begun to shed more light onto the relationships between taxa within the phylum, e.g.^6–11^. Thanks to genetic data for already known, as well as for newly detected species, the discovery and the delimitation of new high rank taxa, such as genera and families, have become more frequent. One example of this trend is the recent erection of the family Richtersiidae Guidetti et al., 2016^11^, which currently comprises four genera: *Adorybiotus* Maucci & Ramazzotti, 1981^12^, *Crenubiotus* Lisi et al., 2020^13^, *Diaforobiotus* Guidetti et al., 2016^11^ and *Richtersius* Pilato & Binda, 1989^14^. Guidetti et al.^11^ explicitly demonstrated that the genera *Adorybiotus*, *Diaforobiotus* and *Richtersius* do not belong to Macrobiotidae Thulin, 1928^15^ and a family of their own was established. However, at the same time, Guidetti et al.^11^ also stressed that the relationships within Richtersiidae need to be clarified with further molecular data as their results indicated a polyphyletic status of the family. More specifically, *Adorybiotus* was shown to be more closely related to Murrayidae than to the other members of Richtersiidae (*Diaforobiotus* + *Richtersius*). Nevertheless, based on morphological similarities, *Adorybiotus* was provisionally included within the Richtersiidae^11^. The same pattern of relatedness within the family was also recovered by the phylogenetic analysis in Guil et al.^16^, based—as in Guidetti et al.^11^—on two conservative ribosomal markers, 18S rRNA and 28S rRNA. Finally, very recently, Lisi et al.^13^ erected a new genus *Crenubiotus* within Richtersiidae based exclusively on morphological evidence. *Crenubiotus* comprises two species: *C. crenulatus* (Richters, 1904)^17^, originally described as a *Macrobiotus* from Svalbard, now the type species for the genus *Crenubiotus*, and *C. revelator* Lisi et al., 2020^13^ discovered in Colombia. Lisi et al.^13^ refined the morphological characters defining the family Richtersiidae as follows: (1) the presence of an additional apophysis on the ventral lamina, (2) the presence of a dorsal apophysis on the buccal tube (reduced in *Crenubiotus* and *Diaforobiotus*), (3) a specific type of claws with a system of internal septa and clearly dentate lunulae on all legs, (4) the presence of cuticular pores (at least in some life stages), and (5) the presence of two macroplacoids in the bulbus.

In this study, we present an upgraded molecular phylogeny of the families Richtersiidae and Murrayidae based on four genetic markers (18S rRNA, 28S rRNA, ITS-2 and COI). New DNA sequences for six species/populations of *Adorybiotus*, *Crenubiotus* and *Diaforobiotus,* as well as two additional species of the family Murrayidae are added to the dataset available from earlier studies. Our results show that *Adorybiotus* and *Crenubiotus* form a clade that is more closely related to the family Murrayidae than to the other two genera of Richtersiidae (*Richtersius* and *Diaforobiotus*). These results, together with evident differences recovered by morphological analysis, led us to the erection of a new family within Macrobiotoidea. Finally, the analysis of a population of *Crenubiotus crenulatus* from the original *terra typica* (Svalbard, Norway) under the integrative taxonomy framework enabled us to redescribe the species and propose a new neotype that will stabilise the taxonomy and will help to uncover the diversity within this recently erected genus.

## Results

### Phylogenetic analysis

**Figure 1:**
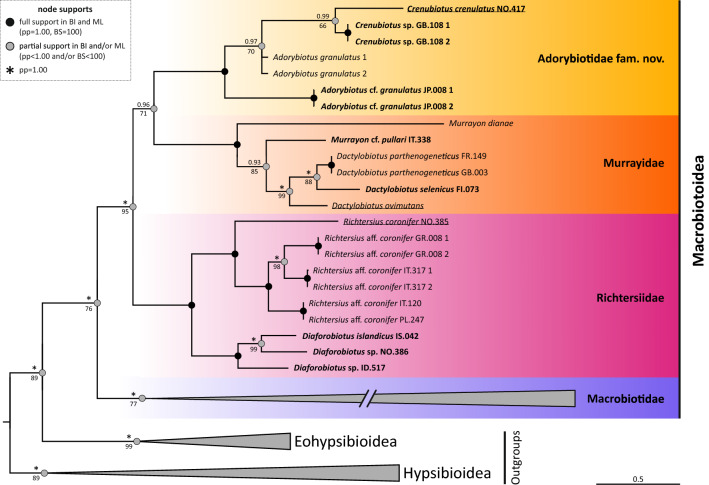
Phylogenetic reconstruction of the superfamily Macrobiotoidea based on concatenated 18S rRNA + 28S rRNA + ITS-2 + COI nucleotide sequences. Topology and branch length of BI reconstruction. Nodes with BI support under 0.70 were collapsed. Values above branches indicate BI posterior probabilities (pp), values below branches indicate ML bootstrap (bs) support. Black circle and no value = full support in both analyses, i.e. 1.00 for BI or 100 for ML; grey circle = node supported in both analyses but not fully in at least one of them (* = full support, BI/ML values lower than 1.00/100 shown). Newly sequenced taxa/populations are bolded. Neotype or type populations are underlined.

We have obtained good quality sequences of all four markers for all sequenced individuals. The phylogenetic reconstruction performed with BI and ML methods (Fig. 1) showed almost identical topologies, with lower support values for the ML tree. The superfamily Macrobiotoidea was recovered monophyletic and composed of four well supported clades (Fig. 1; the following sentences decribe the topology of the tree from the bottom to the top). The first clade represents the family Macrobiotidae, the second clade comprises a part of the family Richtersiidae (*Richtersius* and *Diaforobiotus* but not *Adorybiotus* and *Crenubiotus*), the third clade contains Murrayidae (i.e. *Murrayon* and *Dactylobiotus*), and the fourth clade is composed of *Adorybiotus* and *Crenubiotus*. The last three clades are more closely related to each other than to the family Macrobiotidae. Moreover, the family Richtersiidae is a sister group to Murrayidae + (*Adorybiotus* + *Crenubiotus*). Given the evident phylogenetic and morphological distinctiveness, the *Adorybiotus* + *Crenubiotus* clade is further elevated to the family level (see the next section below for more details). The majority of the genera in the families Richtersiidae, Murrayidae and in the new family Adorybiotidae were retrieved as monophyletic. The only two paraphyletic genera in our reconstruction were *Murrayon* and *Adorybiotus*. In the genus *Murrayon*, *M. dianae* was found to be sister to the *Murrayon* cf. *pullari* + the *Dactylobiotus* clade. A similar topology was found for *Adorybiotus*, with *Adorybiotus* sp. JP.008 being sister to *Adorybiotus granulatus* + *Crenubiotus crenulatus*.

### Description of the new family

Systematic and taxonomic account

- **Phylum:** Tardigrada Doyère, 1840^18^.
- **Class:** Eutardigrada Richters, 1926^19^.
- **Order:** Parachela Schuster et al., 1980^20^ (restored in Morek et al.^21^).
- **Superfamily:** Macrobiotoidea Thulin, 1928^15^.
- **Family:** Adorybiotidae **fam. nov.** Stec, Vecchi & Michalczyk.
- ZooBank: urn:lsid:zoobank.org:act:CC69D220-D0D8-43CA-86AE-E998FB843D6D.
  (Figs. 1, 2, 3, 4, 5, 6, 7, 8, 9, 10, 11, 12).

**Figure 2:**
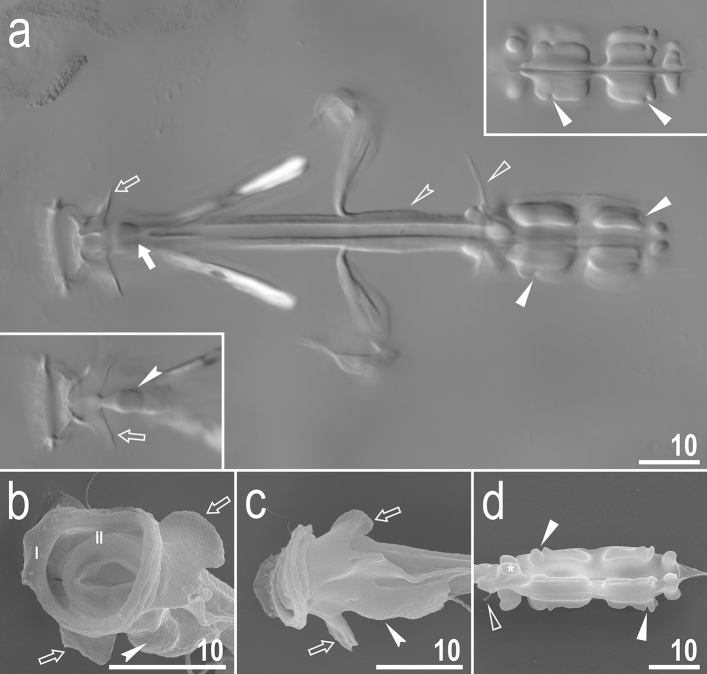
*Adorybiotus* cf. *granulatus* from Japan: buccal apparatus. (**a**) Dorsal projection of the entire buccal apparatus seen in NCM; left lower insert: ventral projection of the anterior portion of the buccal apparatus; right upper insert: ventral projection of the placoids. (**b**) Buccal crown and oral cavity followed by the buccal tube opening, frontal view. (**c**) Buccal crown, lateral view. (**d**) Placoids, dorsal view. The filled arrow indicates the cuticular hook on the T-shaped apophysis, empty arrows indicate the lateral triangular apophysis, filled indented arrowheads indicate the bulbous apophysis at the anterior end of the ventral lamina, filled flat arrowheads indicate constrictions in the macroplacoids, empty flat arrowheads indicate dorsal spikes, Roman numerals indicate the bands of teeth in the oral cavity. Scale bars in μm.

**Figure 3:**
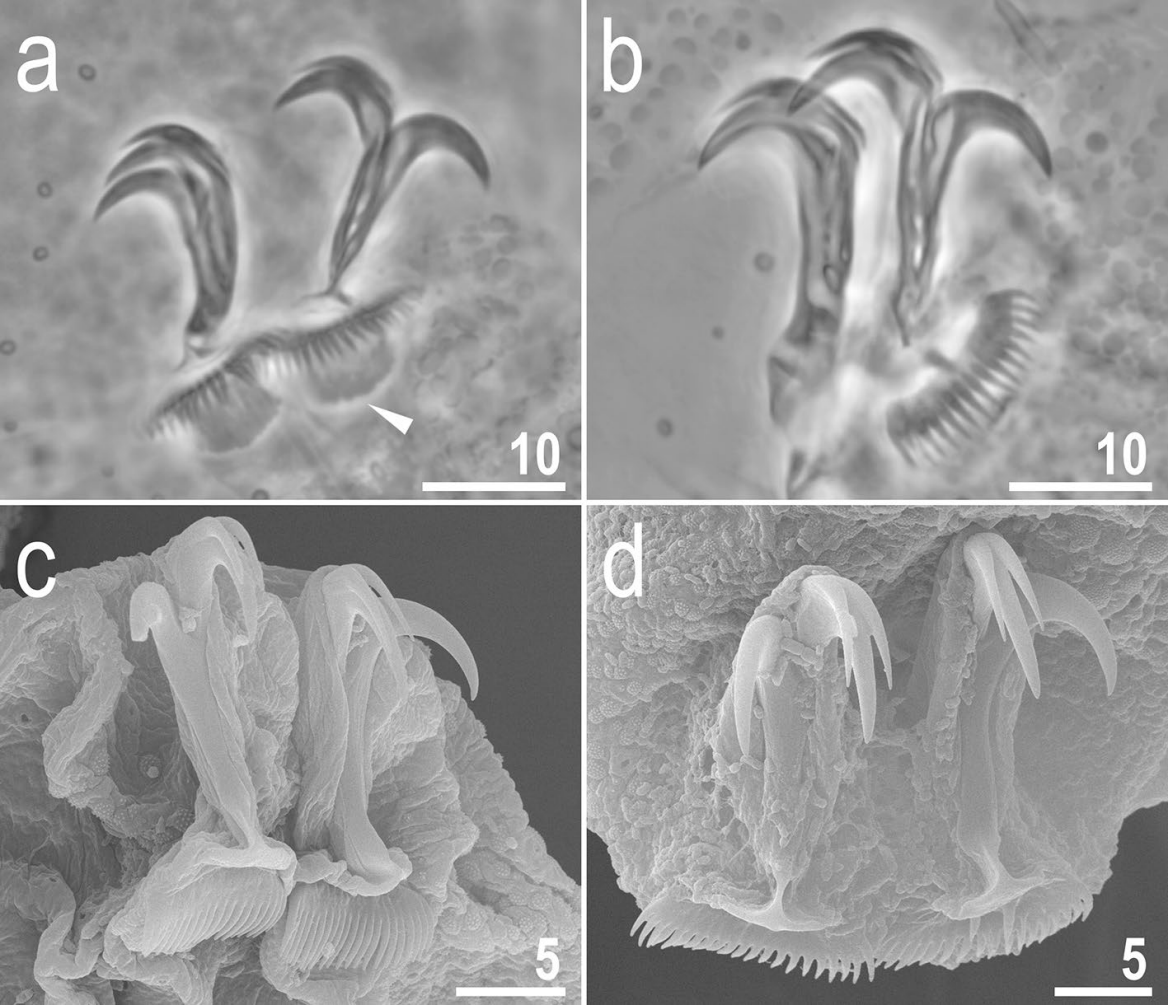
*Adorybiotus* cf. *granulatus* from Japan: claws. (**a**) Claws II (PCM). (**b**) Claws IV (PCM). (**c**) Claws III (SEM). (**d**) Claws IV (SEM). The arrowhead indicates paired cuticular swellings/thickenings under the claws. Scale bars in μm.

**Figure 4:**
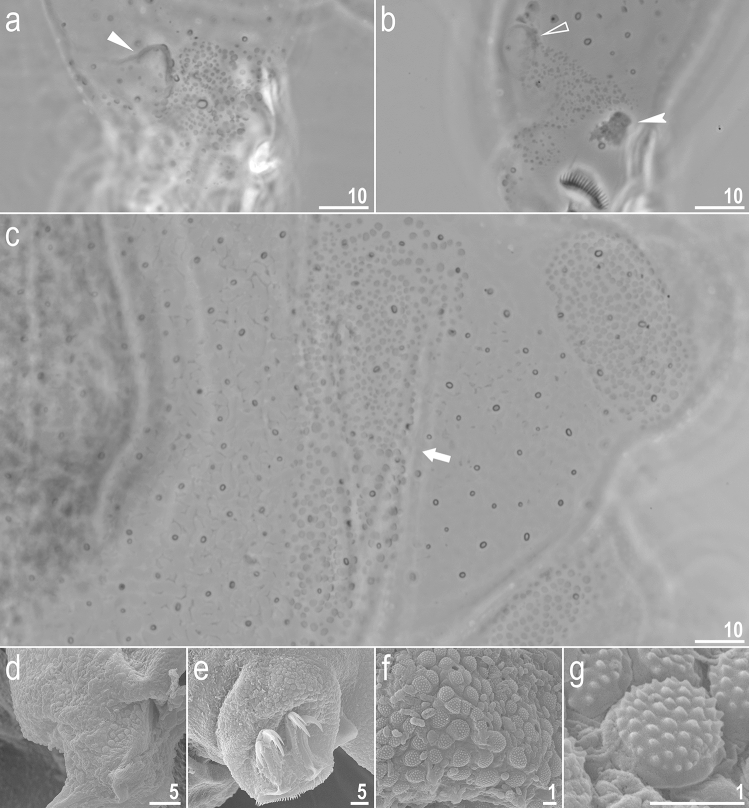
*Adorybiotus* cf. *granulatus* from Japan: cuticular granulation. (**a**) Granulation on the external surface of leg I (PCM). (**b**) Granulation on the internal surface of leg I (PCM). (**c**) Wide granulation band on the caudal end of the body and granulation on legs IV (PCM). (**d**) Granulation on the external surface of leg II (SEM). (**e**) Granulation on leg IV (SEM). (**f**,**g**) Details of the cuticular granulation (SEM). The flat arrow indicates the cuticular bulge/fold on the external leg surface, the empty flat arrowhead indicates the cuticular bulge/fold on the internal leg surface, the filled indented arrowhead indicates paired muscle attachments under claws, the arrow indicates the wide granulation band on the caudal cuticle. Scale bars in μm.

**Figure 5:**
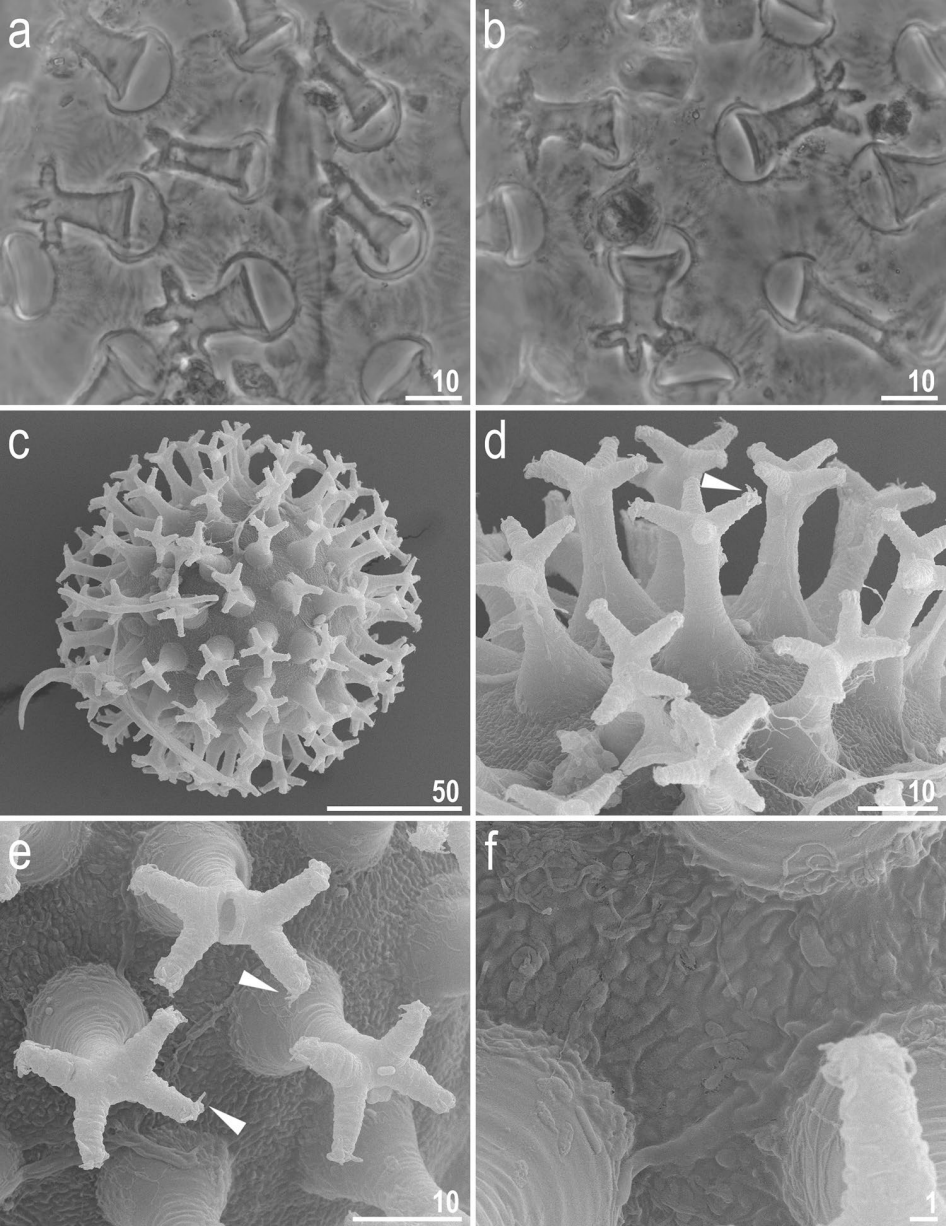
*Adorybiotus* cf. *granulatus* from Japan: eggs morphology. (**a**,**b**) Egg surface and processes seen in PCM. (**c**) Entire egg seen in SEM. (**d**,**e**) Details of egg processes morphology seen in SEM. (**f**) Details of the egg surface between processes seen in SEM. Arrowheads indicate short flexible filaments at the end of multifurcated process apices. Scale bars in μm.

**Figure 6:**
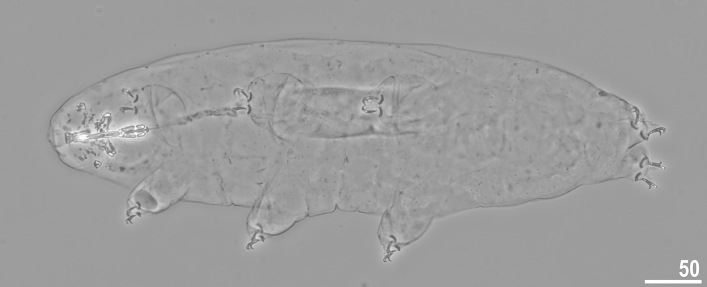
*Crenubiotus crenulatus s.s.* (Richters, 1904) from Spitsbergen: adult habitus, dorso-ventral projection, neotype. Scale bars in μm.

**Figure 7:**
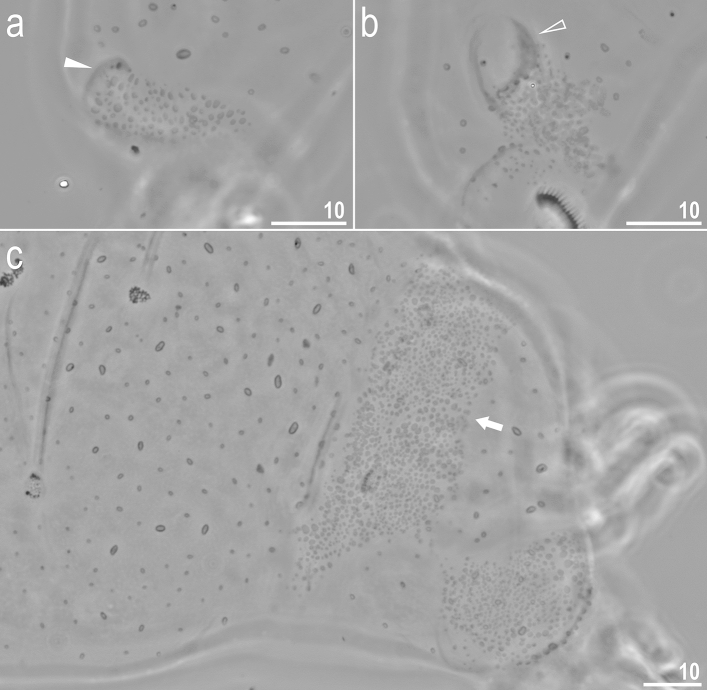
*Crenubiotus crenulatus s.s.* (Richters, 1904) from Spitsbergen: cuticular granulation seen in PCM. (**a**) Granulation on the external surface of leg II. (**b**) Granulation on the internal surface of leg II. (**c**) The wide granulation band on the caudal cuticle and granulation on legs IV. The filled flat arrowhead indicates the cuticular bulge/fold on the external leg surface, the empty flat arrowhead indicates the cuticular bulge/fold on the internal leg surface, the arrow indicates the wide granulation band on the caudal cuticle. Scale bars in μm.

**Figure 8:**
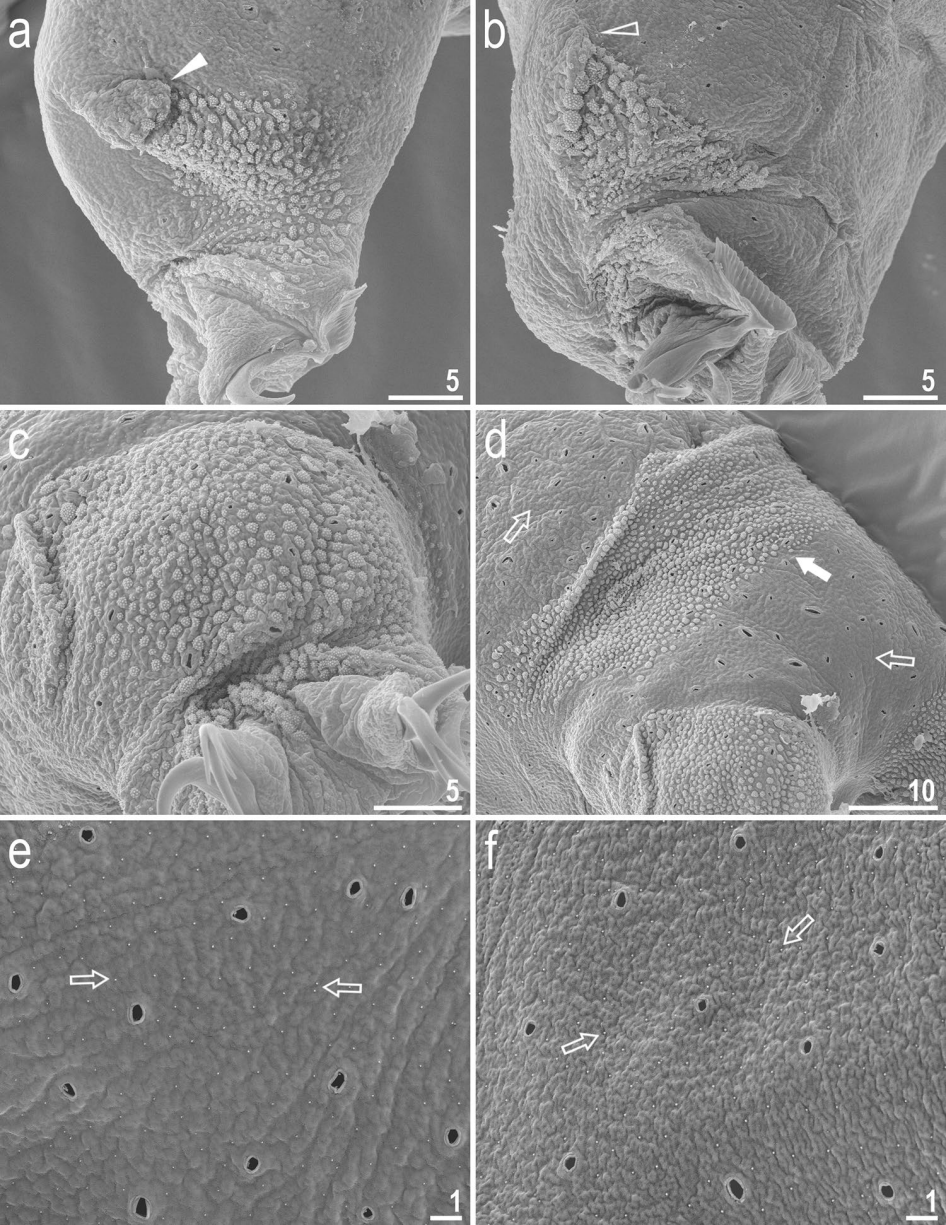
*Crenubiotus crenulatus s.s.* (Richters, 1904) from Spitsbergen: cuticular granulation seen in SEM. (**a**) Granulation on the external surface of leg III. (**b**) Granulation on the internal surface of leg III. (**c**) Granulation on leg IV. (**d**) The wide granulation band on the caudal cuticle and granulation on legs IV. (**e**) Sparse cuticular granulation and pores on the dorsal cuticle. (**f**) Sparse cuticular granulation and pores on the ventral cuticle. The filled flat arrow indicates the cuticular bulge/fold on the external leg surface, the empty flat arrowhead indicates the cuticular bulge/fold on the internal leg surface, the filled arrow indicates the wide granulation band on the caudal cuticle, empty arrows indicate the sparse cuticular granulation. Scale bars in μm.

**Figure 9:**
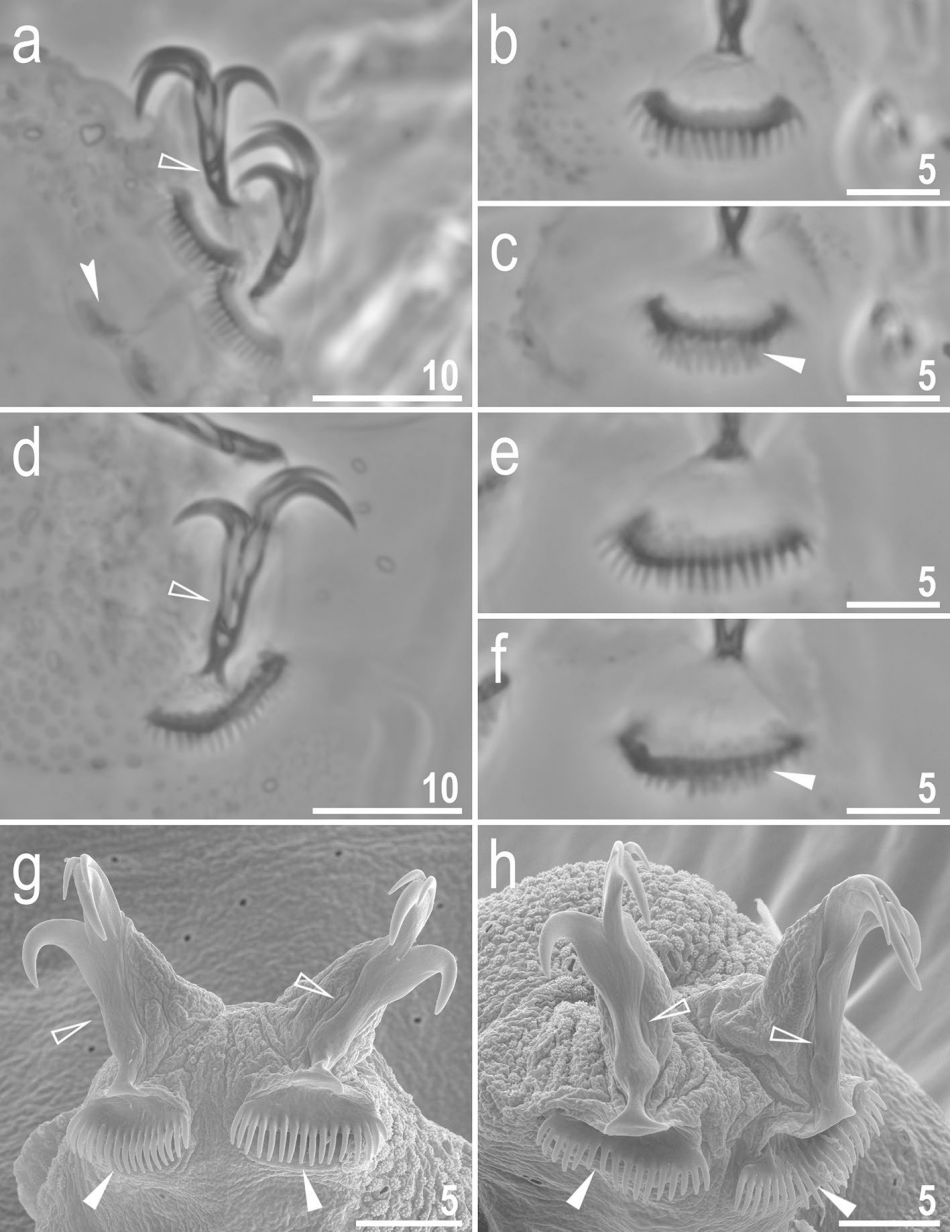
*Crenubiotus crenulatus s.s.* (Richters, 1904) from Spitsbergen: claws. (**a**) Claws I (PCM). (**b**) Lunula II (PCM) (**c**) The cuticular swelling/thickening under lunula II (PCM). (**d**) Claws IV (PCM). (**e**) Lunula IV (PCM). (**f**) The cuticular swelling/thickening under lunula IV (PCM). (**g**) Claws I (SEM). (**h**) Claws IV (SEM). Photos (**b**,**c**) and e–f show the same lunula, respectively. Empty flat arrowheads indicate slight constrictions in the middle of the claws, the filled indented arrowhead indicates double muscle attachments below the claws, filled flat arrowheads indicate cuticular swellings/thickenings under lunulae. Scale bars in μm.

**Figure 10:**
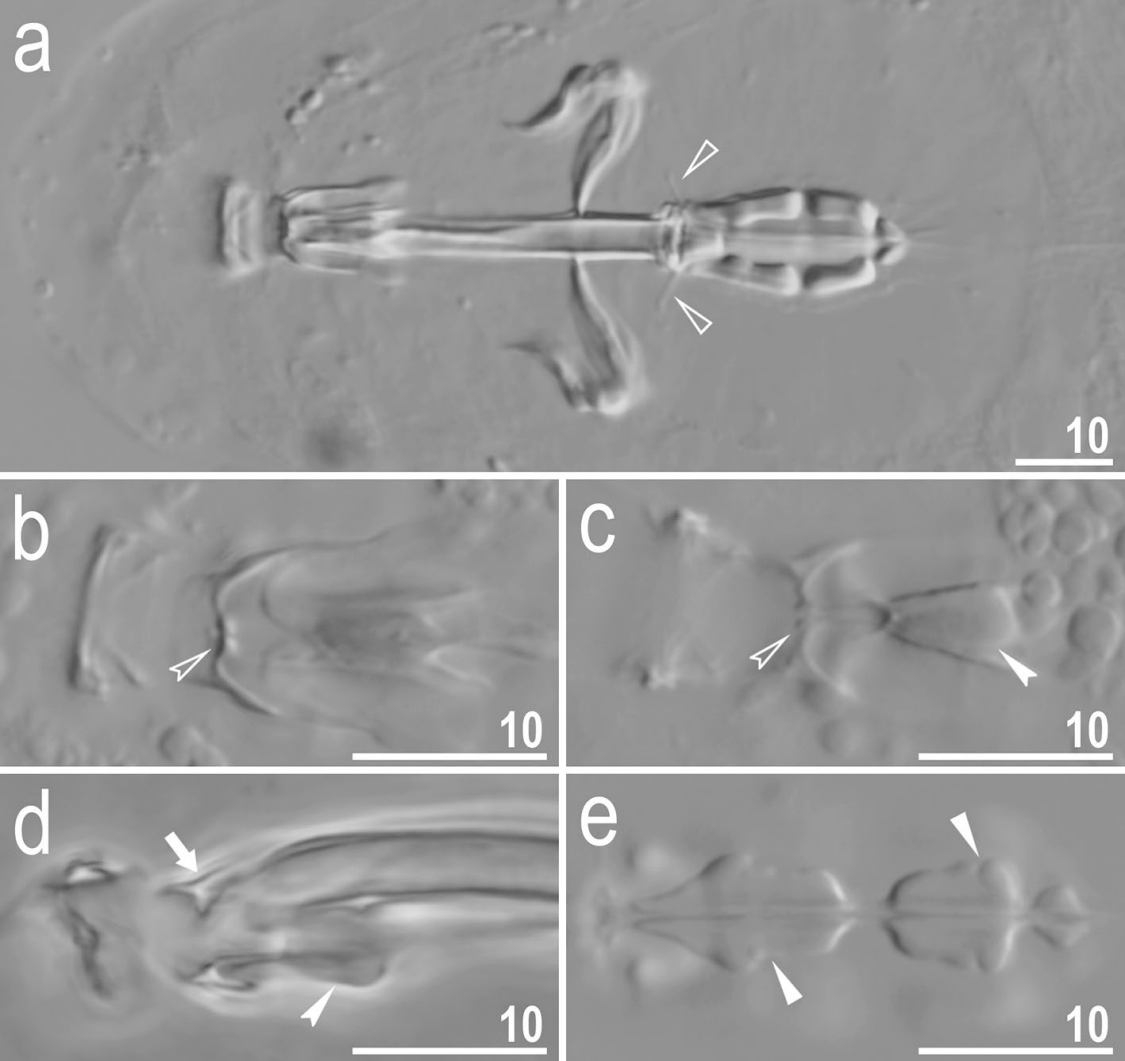
*Crenubiotus crenulatus s.s.* (Richters, 1904) from Spitsbergen: buccal apparatus seen in PCM. (**a**) Dorsal projection of the entire buccal apparatus. (**b**) Dorsal view of the oral cavity armature. (**c**) Ventral view of the oral cavity armature. (**d**) Lateral view of the anterior portion of the buccal apparatus. (**e**) Ventral view of placoids. Empty flat arrowheads indicate dorsal spikes, filled indented arrowheads indicate the ventral thickening/additional ridge on the ventral lamina, empty indented arrowheads indicate the third band of teeth in the oral cavity armature, the arrow indicates the putative dorsal residual apophysis, filled flat arrowheads indicate constrictions in the macroplacoids. Scale bars in μm.

**Figure 11:**
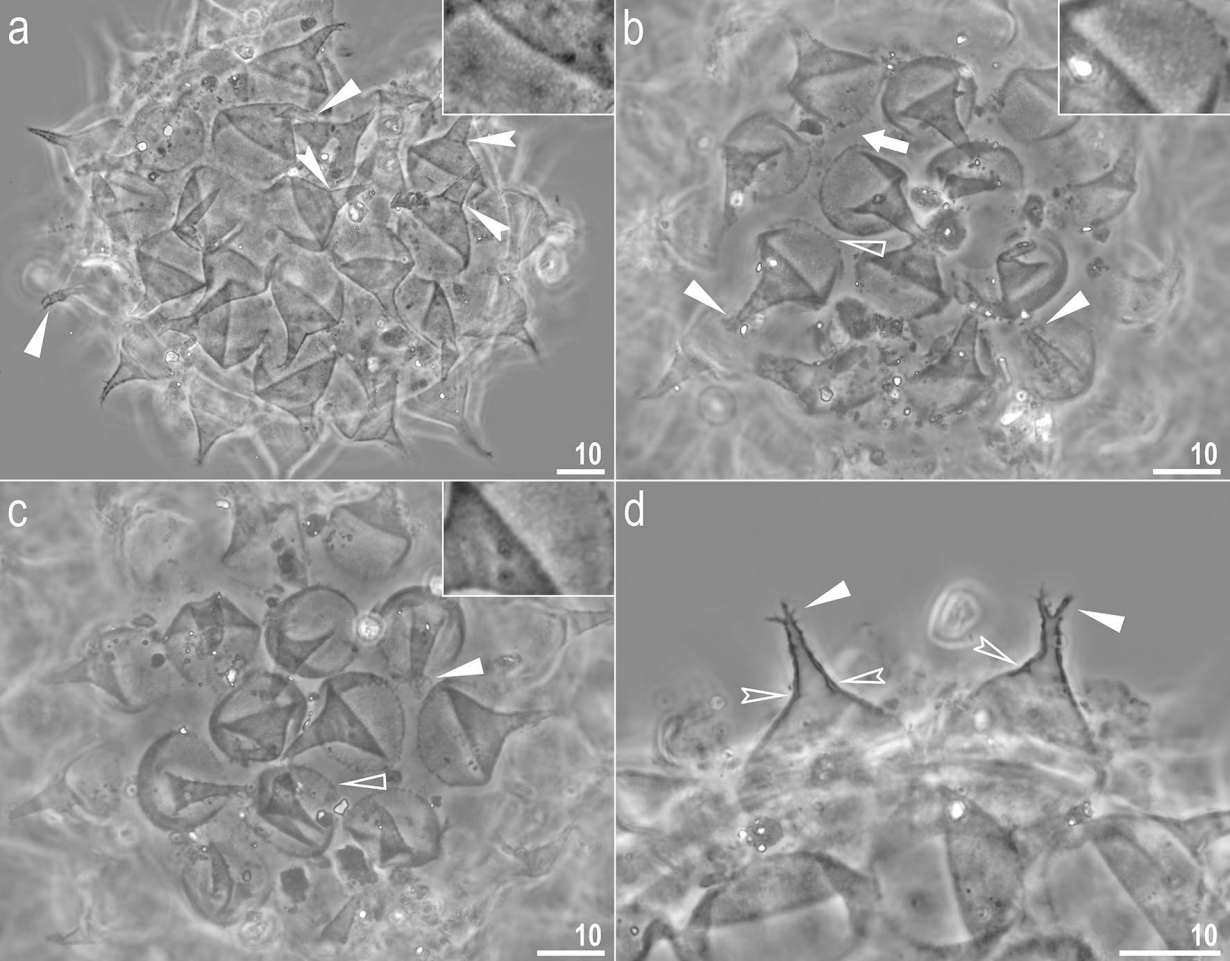
*Crenubiotus crenulatus s.s.* (Richters, 1904) from Spitsbergen: eggs seen in PCM. (**a**–**c**) Details of egg processes and surface under a ×1000 magnification, upper inserts shows details of the ‘reticulation’ present in egg processes of a given egg. (**d**) Midsections of egg processes under a ×1000 magnification. Filled flat arrowheads indicate divided tips of the processes, filled indented arrowheads indicate faint septae, empty flat arrowheads indicate short thickenings/projections around processes bases, empty indented arrowheads indicate thickenings within the processes walls between main and distal parts of the processes, the arrow indicates elongated thickenings/projections around processes the bases. Scale bars in μm, scale bar for inserts in figures a–c are twice as long as those in the main photos.

**Figure 12:**
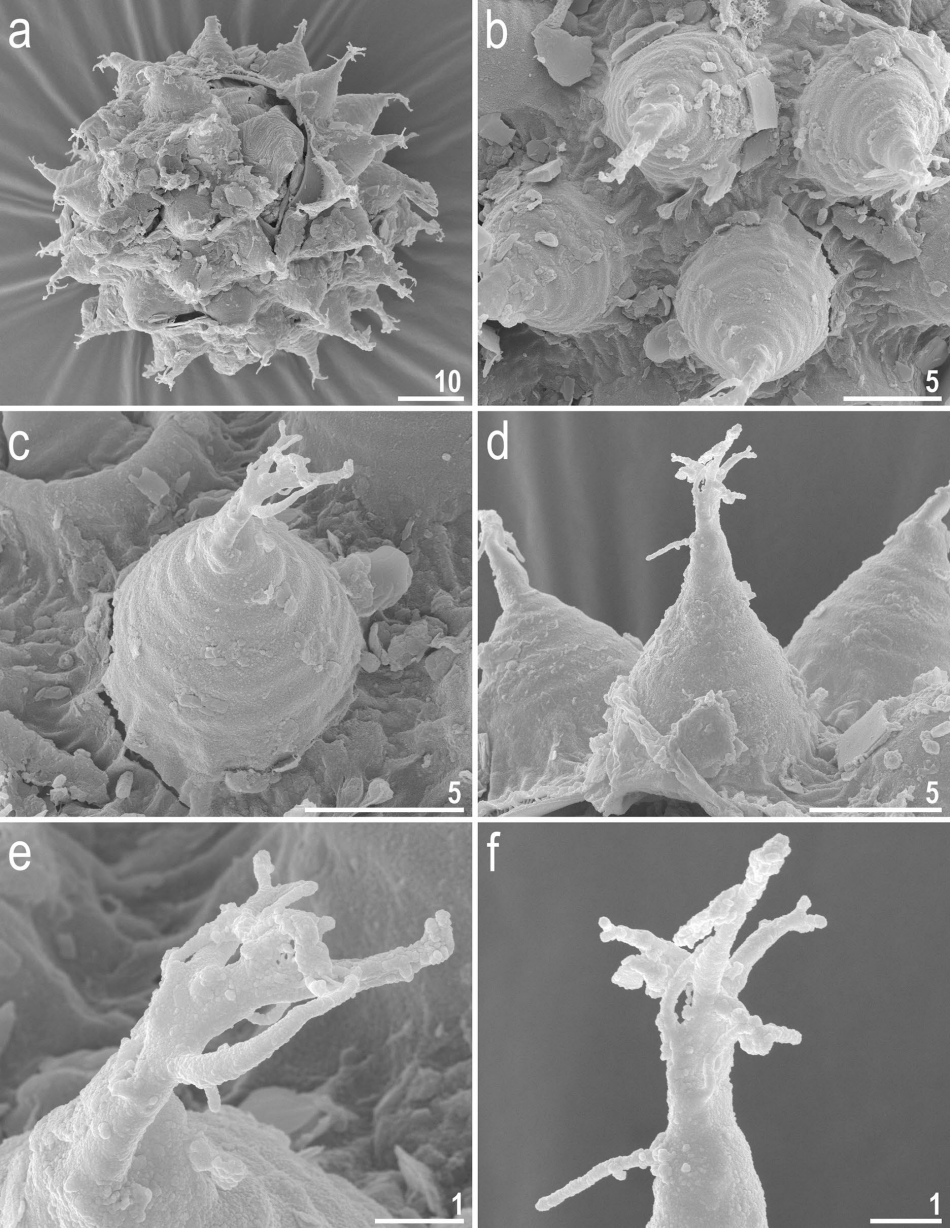
*Crenubiotus crenulatus* (Richters, 1904) from Spitsbergen: eggs seen in SEM. (**a**) Entire egg. (**b**) Details of egg surface. (**c**,**d**) Details of egg processes. (**e**,**f**) Details of egg process apices. Scale bars in μm.

#### Diagnosis:

Tubercles/cushions with aggregations of microgranules on their surfaces present on all legs and on the dorso-caudal cuticle. Cuticular pores present in all instars. Double Y-shaped claws, with the two branches forming an evident common tract of a variable length. Large, comb-like lunulae under claws on each leg, equipped with long and evenly distributed teeth. Buccal tube with the ventral lamina and a cuticular thickening (which can form a large apophysis) on the anterio-dorsal wall of the buccal tube. Two macroplacoids and a microplacoid positioned close to the second macroplacoid in the bulbus. Ornamented eggs without areolation on the surface between the egg processes laid freely to the environment.

#### Type genus:

*Adorybiotus* Maucci & Ramazzotti, 1981^12^.

#### Family composition:

*Adorybiotus* (Figs. 2, 3, 4, 5), *Crenubiotus* Lisi et al., 2020^13^. (Fig. 6, 7, 8,9, 10, 11, 12).

Adorybiotidae **fam. nov.**, by the combination of morphological characters of animals and eggs is unique within the superfamily Macrobiotoidea, and it differs specifically from the family:

- **Macrobiotidae** Thulin, 1928^15^ by: large comb-like lunulae under claws on each leg equipped with long and evenly distributed teeth (lunulae smaller, often without teeth and when equipped with teeth, they are short and not as regularly arranged as in Adorybiotidae **fam. nov.**); and by the system of internal septa within claws on each leg as described by Lisi et al.^13^ (the system of internal septa absent in Macrobiotidae).
- **Richtersiidae** Guidetti et al., 2016^11^ by: the presence of tubercles/cushions with aggregations of microgranules on their surfaces on all legs and on the dorso-caudal cuticle (granulation on legs and on the dorso-caudal cuticle absent in Richtersiidae); the presence of the microplacoid in the bulbus (microplacoid absent in Richtersiidae); and by egg process morphology (processes in the shape of cones with wide bases and very narrow elongated apices or processes with concave bases in the shape of cooling towers and apices divided into 2–4 thick horizontal branches in Adorybiotidae **fam. nov.** *vs* egg process in the shape of elongated, thin, conical spikes in Richtersiidae).
- **Murrayidae** Guidetti et al., 2005^22^ by: cuticular pores (absent in Murrayidae); large comb-like lunulae under claws on each leg equipped with long and evenly distributed teeth (lunulae without teeth in Murrayidae); the system of internal septa within claws on each leg as described by Lisi et al.^13^ (the system of internal septa absent in Murrayidae); the presence of tubercles/cushions with aggregations of microgranules on their surfaces on all legs and on the dorso-caudal cuticle (only regular granulation present but tubercles/cushions absent in Murrayidae); and by claw morphology (the common tract of the claw longer than the half of the entire claw height in Adorybiotidae **fam. nov.** *vs* the common tract of the claw shorter than the half of the entire claw height in Murrayidae).

#### Genus:

*Crenubiotus* Lisi et al., 2020^13^.

***Crenubiotus crenulatus*** (Richters, 1904)^17^.

(Tables 1, 2, Figs. 6, 7, 8,9, 10, 11, 12).

**Table 1:** Measurements [in µm] of selected morphological structures of individuals from the neotype population of *C*. *crenulatus s.s.* (Richters, 1904) mounted in Hoyer’s medium (N—number of specimens/structures measured, RANGE refers to the smallest and the largest structure among all measured specimens; SD—standard deviation).

Character	N	Range		Mean		SD		Neotype	
		µm	*pt*	µm	*pt*	µm	*pt*	µm	*pt*
Body length	18	380–584	*897–1361*	441	*1099*	54	*104*	515	*1223*
**Buccal tube**									
Buccal tube length	18	33.6–42.9	–	40.1	*–*	2.4	*–*	42.1	*–*
Stylet support insertion point	18	24.4–31.7	*71.9–74.6*	29.6	*73.7*	1.9	*0.7*	31.0	*73.6*
Buccal tube external width	18	3.6–5.0	*9.2–12.1*	4.3	*10.8*	0.4	*0.8*	4.5	*10.7*
Buccal tube internal width	18	1.8–2.8	*5.0–6.6*	2.3	*5.7*	0.3	*0.5*	2.5	*5.9*
Ventral lamina length	16	19.8–25.3	*53.4–61.7*	23.1	*57.5*	1.6	*2.3*	24.6	*58.4*
**Placoid lengths**									
Macroplacoid 1	18	7.5–11.0	*21.8–27.2*	9.5	*23.7*	1.0	*1.7*	9.5	*22.6*
Macroplacoid 2	18	4.9–7.4	*13.0–17.6*	6.3	*15.7*	0.6	*1.1*	6.6	*15.7*
Microplacoid	18	2.0–3.0	*5.3–7.0*	2.5	*6.2*	0.3	*0.5*	2.7	*6.4*
Macroplacoid row	18	13.8–20.3	*39.0–48.2*	17.5	*43.7*	1.7	*2.6*	18.5	*43.9*
Placoid row	18	16.5–23.2	*45.8–55.1*	20.5	*51.1*	1.7	*2.6*	22.2	*52.7*
**Claw 1 heights**									
External base	8	5.7–9.5	*14.5–22.1*	7.7	*18.8*	1.2	*2.5*	?	*?*
External primary branch	16	8.6–14.0	*21.9–32.6*	11.5	*28.4*	1.5	*2.6*	12.0	*28.5*
External secondary branch	12	6.0–11.0	*15.3–25.6*	8.8	*21.7*	1.4	*2.7*	9.4	*22.3*
External base/primary branch (*cct*)	8	*64.8*–*70.4*	–	*67.5*	*–*	2.1	*–*	?	*–*
Internal base	8	5.4–8.6	*13.7–20.7*	7.6	*18.4*	1.0	*2.1*	8.0	*19.0*
Internal primary branch	16	8.4–13.3	*21.4–31.3*	11.1	*27.6*	1.4	*2.5*	11.9	*28.3*
Internal secondary branch	14	6.0–9.8	*15.3–23.2*	8.3	*20.8*	1.2	*2.2*	9.2	*21.9*
Internal base/primary branch (*cct*)	8	*63.9–70.4*	–	*66.7*	*–*	2.4	*–*	*67.2*	*–*
**Claw 2 heights**									
External base	15	6.3–10.1	*16.0–24.9*	8.4	*20.9*	1.2	*2.3*	8.6	*20.4*
External primary branch	17	9.6–15.8	*24.7–36.8*	12.5	*31.0*	1.7	*3.1*	13.3	*31.6*
External secondary branch	15	6.4–11.7	*16.3–27.7*	9.4	*23.5*	1.5	*3.0*	?	*?*
External base/primary branch (*cct*)	15	*63.9*–*70.1*	–	*67.1*	*–*	2.2	*–*	64.7	*–*
Internal base	13	6.0–9.8	*15.5–22.8*	8.1	*20.2*	1.2	*2.2*	?	*?*
Internal primary branch	16	8.7–15.2	*22.4–35.4*	11.8	*29.5*	1.7	*3.1*	13.1	*31.1*
Internal secondary branch	16	6.5–11.5	*16.5–26.8*	9.4	*23.4*	1.5	*2.8*	10.4	*24.7*
Internal base/primary branch (*cct*)	13	*64.5*–*70.2*	–	*68.5*	*–*	1.7	*–*	?	*–*
**Claw 3 heights**									
External base	14	6.4–10.5	*16.3–25.9*	8.7	*21.6*	1.2	*2.4*	9.4	*22.3*
External primary branch	18	9.4–16.1	*23.9–38.0*	12.8	*31.9*	1.8	*3.5*	14.2	*33.7*
External secondary branch	17	8.3–12.5	*22.4–30.9*	10.1	*25.2*	1.2	*2.4*	9.9	*23.5*
External base/primary branch (*cct*)	14	*65.4*–*69.4 s*	–	*67.9*	*–*	1.2	*–*	*66.2*	*–*
Internal base	13	6.0–10.0	*15.3–24.4*	8.5	*21.0*	1.2	*2.4*	9.1	*21.6*
Internal primary branch	18	9.0–15.5	*22.9–36.1*	12.2	*30.4*	1.7	*3.3*	13.5	*32.1*
Internal secondary branch	18	7.2–12.0	*18.3–28.2*	9.6	*23.8*	1.3	*2.5*	9.9	*23.5*
Internal base/primary branch (*cct*)	13	*62.6*–*70.7*	–	*67.6*	*–*	2.2	*–*	*67.4*	*–*
**Claw 4 heights**									
Anterior base	15	6.7–10.5	*17.0–25.9*	9.0	*22.3*	1.2	*2.3*	9.3	*22.1*
Anterior primary branch	18	9.8–16.3	*24.9–38.3*	13.5	*33.7*	1.8	*3.5*	14.2	*33.7*
Anterior secondary branch	16	7.1–12.0	*18.1–29.4*	10.3	*25.6*	1.4	*2.8*	10.8	*25.7*
Anterior base/primary branch (*cct*)	15	*64.3*–*70.8*	–	*66.4*	*–*	2.0	*–*	*65.5*	*–*
Posterior base	15	6.7–11.1	*17.0–27.4*	9.7	*23.7*	1.2	*2.7*	10.5	*24.9*
Posterior primary branch	18	9.9–16.9	*25.2–39.4*	14.1	*35.1*	1.9	*3.6*	16.1	*38.2*
Posterior secondary branch	12	7.7–12.7	*19.6–31.4*	11.1	*27.2*	1.5	*3.4*	11.9	*28.3*
Posterior base/primary branch (*cct*)	15	*63.3*–*70.7*	–	*66.6*	*–*	2.2	*–*	*65.2*	*–*

**Table 2:** Measurements [in µm] of selected morphological structures of the eggs from the neotype population of *C*. *crenulatus s.s.* (Richters, 1904) mounted in Hoyer’s medium (N—number of eggs/structures measured, RANGE refers to the smallest and the largest structure among all measured specimens; SD—standard deviation).

Character	N	Range	Mean	SD
Egg bare diameter	4	88.5–93.9	92.2	2.5
Egg full diameter	4	122.4–126.5	125.2	1.9
Process height	42	12.2–21.0	16.4	2.1
Process base width	42	9.4–17.1	12.8	1.9
Process base/height ratio	42	*56%–98%*	*78%*	*10%*
Inter-process distance	30	2.1–4.1	2.9	0.5
Number of processes on the egg circumference	4	15–17	16.3	1.0

*Macrobiotus crenulatus* Richters, 1904^17^; *Macrobiotus dentatus* Binda, 1974^23^.

#### Material examined:

39 animals, and 22 eggs. Specimens mounted on microscope slides in Hoyer’s medium (28 animals + 16 eggs), fixed on SEM stubs (10 + 6), processed for DNA sequencing (1 animal).

#### Neotype locality:

78°12′58.4"N, 15°20′45.1"E; 53 m asl: Norway, Svalbard, Spitsbergen, Lower part of the Bjørndalen (Bjørn valley; Nordenskiöld Land); moss from tundra on the valley slope; coll. 23.07.2016 by Wojciech Maciejowski.

#### Type depositories:

Neotype (slide NO.429.06 with 3 neoparatypes) and remaining 18 neoparatypes (slides: NO.429.*, where the asterisk can be substituted by any of the following numbers 03, 07–09) and 14 eggs (slides: NO.429.*: 01–05) and SEM stub 20.01 are deposited at the Institute of Zoology and Biomedical Research, Jagiellonian University, Gronostajowa 9, 30–387, Kraków, Poland. Six neoparatypes (slide NO.429.11) and two eggs (slide NO.429.10) are deposited in the Pilato and Binda collection at the University of Catania, Italy.

### Redescription of *Crenubiotus crenulatus* (Richters, 1904)

*Animals (measurements and statistics in* Table 1*):* When alive, body almost transparent in juveniles and yellowish in adults; after fixation in Hoyer’s medium body transparent (Fig. 6). Eyes present, visible also in specimens mounted in Hoyer’s medium. Body cuticle with larger elliptical (0.8–2.0 µm in diameter) and smaller circular (0.3–0.8 µm) pores distributed randomly on the entire body cuticle with the largest elliptical pores being present in the dorso-cephalic and dorso-caudal cuticle (Figs. 7a–c, 8a–f). Patches of dense very visible granulation that comprises of small tubercles/cushions and aggregations of microgranules on their surfaces present on all legs and on the dorso-caudal cuticle (Figs. 7a–c, 8a–d). These tubercles are slightly less developed and evident compared to *Adorybiotus* (Fig. 4a–g) but still obvious (Figs. 7a–c, 8a–d). On legs I–III, the dense granulation comprises of two patches on the external and internal leg surfaces respectively, that are connected with each other by a narrower band of granulation extending from them on the proximal leg surface (Figs. 7a,b, 8a,b). Two pulvini are present on each leg I–III, one on the external and the other—on the internal leg surface (Figs. 7a,b, 8a,b). On legs IV, dense granulation covers evenly the dorsal and lateral leg surfaces (Figs. 7c, 8c,d). This dense granulation is also present as a wide granulation band on the caudal cuticle that extends across the terminal body segment from the left body side, through dorsal surface, to the right side (Figs. 7c, 8d). Beside these dense granulation patches, very fine granulation is present and evenly distributed on the entire body surface but visible only under SEM (Fig. 8a–f). *Remarks*: The large elliptical pores reported by Lisi et al.^13^ as located laterally to the mouth in *C. revelator* are also present in *C. crenulatus*, however their exact position and shape could not be determined as the mouth was retracted in almost all analysed specimens.

Claws slender, of the Richtersiidae type. Primary branches with distinct accessory points, a long, constricted in the middle, common tract with a system of internal septa, and with an evident stalk connecting the claw to the lunula (Fig. 9a–h). The common tract apparently longer than the half of the entire claw height (Fig. 9a,d,g–h). Large, comb-like, triangular lunulae with long and evenly distributed teeth present on all legs (Fig. 9a–h). Under PCM, the lower portion of the lunulae just above the dentation is evidently darker and visible as a dark arc (Fig. 9a–b, d–e). The lunulae are curved and clamped around the cuticular swelling/thickening present under them (Fig. 9g–h) what is well visible in SEM whereas in PCM on the lower focal plane it is visible as a darkening beneath the lunules (Fig. 9c,f). Paired muscle attachments present just below lunulae on legs I–III (Fig. 9a).

Mouth antero-ventral. Bucco-pharyngeal apparatus of modified “*Macrobiotus* type” (Fig. 10a), i.e. with ten peribuccal lamellae, a rigid buccal tube with the ventral lamina which is provided with an additional ventral thickening in its anterior portion, that appears as an elongated trapezoidal structure pointing towards the mouth opening in the ventral view (Fig. 10c) and is visible as a ridge in the lateral view (Fig. 10d). Based on LCM observations, the oral cavity armature is poorly developed and composed only of the third band of teeth (Fig. 10b,c). The first and the second band of teeth are absent or not visible under LCM (Fig. 10b,c). The teeth of the third band are located within the posterior portion of the oral cavity, anteriorly to the buccal tube opening (Fig. 10b,c). The third band of teeth is divided into the dorsal and the ventral portion. Under LCM, both the dorsal and the ventral portions are seen as two distinct transverse ridges and each of them forms a globular thickening at the medial extremity (Fig. 10b,c). Median teeth absent (Fig. 10b,c). Bulbus spherical (Fig. 10a), with triangular apophyses, three anterior cuticular spikes (typically only two are visible in any given plane) and two rod-shaped macroplacoids (2 < 1) and a microplacoid positioned close to the second macroplacoid (Fig. 10a,e). The first macroplacoid is anteriorly narrowed and constricted in the middle whereas the second has a sub-terminal constriction (Fig. 10e).

*Eggs (measurements and statistics in* Table 2*):* Laid freely, yellowish, spherical with conical processes with elongated apices and egg surface without areolation (Figs. 11a–d, 12a–f). The elongated process apices are often multifurcated into short flexible filaments (Figs. 11a–d, 12a–f). These elongated distal portions of the processes seem to be sometimes separated from the lower portion of the process by a faint septum (Fig. 11a) but this is caused a circular thickening on the inner process wall (Fig. 11d). The labyrinthine layer between the process walls is visible as a very faint reticular pattern with circular margins under LCM (Fig. 11a–d). The faint meshes are visible in the lower part of the processes but are not visible in the elongated upper part (Fig. 11a–d). The entire surface of processes is smooth under SEM (Fig. 12a–f). Processes attached to the egg by a ring of short thickenings, seen as dark projections visible only under LCM, which gives the process bases a jagged appearance (Fig. 11b–c). Only rarely some these projections might be elongated making the impression of connection between two neighbouring processes (Fig. 11b), however this character should be treated with a dose of caution as all the eggs were covered with debris, thus these thin connectors may be an artefact. Besides these structures, egg surface between processes appears as smooth under LCM (Fig. 11a–d), whereas it is slightly wrinkled in SEM (Fig. 12a–e).

### Reproductive mode

The examination of 28 adults freshly mounted in Hoyer’s medium revealed no testes or spermathecae filled with spermatozoa, which suggests that the species is (at least facultatively) parthenogenetic.

### DNA sequences

We obtained sequences for all four of the above-mentioned molecular markers from one of the two individuals destined for DNA extraction and sequencing, which are as follow: 18S rRNA (GenBank: MT812474), 994 bp long; 28S rRNA (MT812468), 735 bp long; ITS-2 (MT812606), 398 bp long; COI (MT808079), 629 bp long.

### Phenotypic differential diagnosis

To date, the genus *Crenubiotus* comprises only two species: the nominal *C. crenulatus* and an extremely similar species, *C. revelator*, recently described from Colombia. Despite the overall similarity, *C. crenulatus* differs from *C. revelator* by: the absence of the circular median tooth in the ventral portion of the third band of teeth in the oral cavity (the median tooth present in *C. revelator*), the presence of only two lateral teeth in the ventral portion of the third band of teeth, which have thickenings in their medial extremity that resemble circular teeth (the teeth without thickenings in *C. revelator*), a shorter ventral lamina (19.8–25.3 µm [53.4–61.7] in *C. crenulatus* vs. 12.1–18.5 µm [44.5–50.7] in *C. revelator*), larger eggs (ranges of full and bare diameter for four *C. crenulatus* eggs: 122.4–126.5 µm and 88.5–93.9 µm vs. 97.8 and 78.1 µm of the sole known egg of *C. revelator*).

## Discussion

By the analysis of both morphological and molecular data, we explicitly demonstrated the presence of a previously unrecognised phyletic lineage within the superfamily Macrobiotoidea which comprises two genera, *Adorybiotus* and *Crenubiotus*, for which genetic data were extremely limited or absent. To accommodate the phylogenetic and morphological distinctiveness of this group from the remaining three families within the Macrobiotoidea, we erected the new family Adorybiotidae **fam. nov.** Furthermore, in order to enhance taxonomic studies on the recently erected genus *Crenubiotus* and stabilise its nomenclature, we provided an integrative redescription of *C. crenulatus* based on the population from original *terra typica* and replaced the existing, inadequate neotype with the new one that is associated with DNA barcodes.

Two genera analysed in this study, *Murrayon* in the family Murrayidae and *Adorybiotus* in Adorybiotidae **fam. nov.**, appear to be paraphyletic. As already shown by Bertolani et al.^6^, *Murrayon* cf. *pullari* IT.338 is more closely related to *Dactylobiotus* than to *Murrayon dianae*. However, the paraphyly should be treated with great caution because the *M. dianae* branch is exceptionally long, thus it could lead to topological artefacts. The unbalanced sequencing may also be the cause behind the paraphyly of *Adorybiotus*, as only the 18S rRNA was sequenced for *Adorybiotus granulatus* in Bertolani et al.^6^, in contrast to the other analysed populations of the Adorybiotidae **fam. nov.**, for which a complete set of four markers was available. Thus, a better sampling within *Murrayon* and *Adorybiotus*, both in terms of the number of species and sequenced markers, is needed to verify the phyletic relationships within the two genera.

As was pointed out in the Introduction, thanks to the easier acquisition of genetic data and their use in phylogenetic studies, the relationships between major evolutionary lineages within the phylum Tardigrada are being gradually resolved. The increasing popularity of integrative taxonomy also contributed to the recognition of considerable and yet undescribed species diversity within this animal group, e.g.^10,11,24–26^. The recent years showed several times that old, outdated and inadequate tardigrade species descriptions or redescription are often the major obstacle in resolving the systematics within genera and species complexes^24,25,27–31^. In the families Richtersiidae and Adorybiotidae **fam. nov.**, type species for all genera were described in the beginning of the twentieth century and are insufficient in details under modern standards of tardigrade taxonomy, while the original type series do not exist. However, *Richtersius coronifer* (Richters, 1903)^32^, the nominal species for the genus *Richtersius*, was recently redescribed by Stec et al.^31^ under the integrative taxonomy framework. Although a neotype for this species was previously established by Maucci and Ramazzotti^12^, its designation and redescription have been questioned and considered as an impediment for *Richtersius* taxonomy and nomenclature^31,33^. Thus, Stec and Michalczyk^33^ have formally designated a new neotype based on a population from the original *locus typicus* integratively examined in Stec et al.^31^. Importantly, *Crenubiotus crenulatus* (Richters, 1904)^17^, the type species of its genus, suffers from a similar problem. The species was originally described from the Svalbard Archipelago (specifically from Smeerenburg on Spitsbergen), where it seems to be a common element of the tardigrade fauna, e.g.^34–38^. Nonetheless, the diagnosis of this species has been questioned for many years, specifically concerning its possible synonymy with *Macrobiotus echinogenitus* Richters, 1904, also originally described from the Svalbard Archipelago^13,17,39^. The issue has been clarified to some extent by Binda^40^, who communicated that the types of *C. crenulatus* and *M. echinogenitus* are lost and therefore she redescribed and established neotypes for both those species. Recently, Lisi et al.^13^ provided further morphological details of the *C. crenulatus* neotype established by Binda^40^ in order to differentiate it from a new species from Colombia. Nevertheless, there are some more issues regarding the 1988 neotype designation that have to be stressed and appropriate actions should be undertaken. First of all, the neotype of *C. crenulatus* established by Binda comes from Italy (Valtellina) which is almost 3800 km away from the original *locus typicus.* This neotype locality designation does not comply with the International Code of Zoological Nomenclature (Article 75.3.6) that states that the neotype should came as nearly as practicable from the original type locality and, where relevant, from the same geological horizon or host species as the original name-bearing type^41^. This requirement was in force already when Binda^40^ made her designation^42^. Second, the neotype series is in a bad condition, which prevents a detailed morphological characterisation of the species^13^. Finally, taking into consideration the morphological similarity between *C. crenulatus* and *C. revelator*, which has been highlighted in this study and in Lisi et al.^13^, the future taxonomic studies on the genus *Crenubiotus* will be challenging without the use of DNA barcodes. Therefore, in order to stabilise the taxonomy and nomenclature within *Crenubiotus*, in agreement with to the International Code of Zoological Nomenclature, we established a new neotype from a population found at 180 km from the original *locus typicus*, on the same archipelago. With an integrative redescription that comprises detailed morphological data and associated DNA barcodes, future species identification will be much less problematic that it would have been with Binda types from northern Italy. Furthermore, as the same rule of ICZN that was mentioned above was violated when establishing the neotype of *M. echinogenitus* based on the population from Algeria, which is ca. 5500 km away from the Svalbard Archipelago, we propose to consider this designation as invalid too. As the original description of *M. echinogenitu*s from Richters (1903)^32^ is vague and there has been confusion around its identity^40^, we suggest caution in assigning an individual to this species until it is redescribed with material from the original *locus typicus*.

As obstacles in the form of incomplete and outdated descriptions of the type species for *Richtersius* and *Crenubiotus* (Richtersiidae and Adorybiotidae **fam. nov.**, respectively) have been now removed by Stec et al.^31^ and this study, respectively, attention should be paid to similar issues in the remaining two genera, *Adorybiotus* and *Diaforobiotus*. In this study, we presented some details of *Adorybiotus* morphology and genetics, but they were all based on an undetermined species from Japan that cannot be confidently identified until *Adorybiotus granulatus* (Richters, 1903)^32^, the type and the only species of the genus, is redescribed by means of integrative taxonomy. The species was described originally from Norway (Merok), and later redescribed by Maucci and Ramazzotti^12^ based also on a population from Norway (Steinkjer). Importantly, however, this designation can be questioned as Maucci and Ramazzotti^12^ did not fulfil properly the condition given in Article 75.3.4 of the Code^41^, which requires mentioning the “reasons for believing the name-bearing type specimen(s) (i.e. holotype, or lectotype, or all syntypes, or prior neotype) to be lost or destroyed, and the steps that had been taken to trace it or them” (this requirement was also in force already when Maucci and Ramazzotti^12^ made their designation^42^). This opens up the possibility of an extensive taxonomic study on *A. granulatus* that would result in an integrative redescription and a designation of a new neotype, when a suitable population from central Norway is found. The genus *Diaforobiotus* comprises currently only two subspecies *D. islandicus islandicus* (Richters, 1904)^43^ (the nominal subspecies) and *D. islandicus nicaraguensis* (Séméria, 1985)^44^. For years, numerous records of *D. islandicus islandicus* accumulated in the literature from localities all over the world^45–48^ but it has been demonstrated by Guidetti et al.^11^ as well as by this study that the genus definitely comprises more than one species. Nonetheless, since the types of *D. islandicus islandicus* do not exist and morphological details of the species are unknown, we consider descriptions of new taxa highly hazardous until the type species is redescribed by means of integrative taxonomy. The *Diaforobiotus* population from Iceland analysed in this study is a suitable candidate for the neotype population, which together with two other populations from Norway and Indonesia will be revised by us in the near future and published as an integrative revision of the genus. Such integrative redescriptions of the type taxa have opened and will continue to open the windows for describing species diversity within genera or species groups/complexes, further contributing to our understanding of evolution of microscopic invertebrates, including tardigrades.

## Material and methods

### Samples and specimens

To reconstruct the phylogeny of Richtersiidae and Murrayidae, along with already published data, we analysed eight new populations representing eight species isolated from moss or pond sediment samples collected from eight distinct localities (see Table 3 for details). In our study, by a population we mean a group of conspecific individuals found in a single sample. All samples were processed following a protocol described in detail in Stec et al.^49^.

**Table 3:** Information on moss samples with the species/populations sequenced in the present study.

Sample/population code	Species	Coordinates and altitude	Locality	Collector
JP.008	*Adorybiotus* cf. *granulatus*	36°03′31′′ N 138°20′43′′ E 2127 m asl	Japan, Northern Mt. Yatugatake, Mugikusa Pass,	Atsushi Suzuki
GB.108	*Crenubiotus* sp.	58°54′37.77′′ N 3°22′44.01′′ W 383 m asl	Scotland, Hoy	Brian Blagden
NO.429^a^	*Crenubiotus crenulatus*	78°12′58.4′′ N 15°20′45.1"E 53 m asl	Norway, Svalbard, Spitsbergen, Lower part of the Bjørndalen valley (Nordenskiöld Land)	Wojciech Maciejowski
FI.073	*Dactylobiotus selenicus*	62°13′50.23′′ N 25°44′29.53′′ E 82 m asl	Finland, Jyväskylä, Jyväsjärvi Lake,	Matteo Vecchi
ID.517	*Diaforobiotus* sp.	1°51′20′′ S 120°19′25′′ E 1331 m asl	Indonesia, Lore Lindu, Bada Lembah	Artur Oczkowski & Piotr Gąsiorek
IS.042^b^	*Diaforobiotus islandicus islandicus*	63°52′53′′ N 22°27′21′′ W 44 m asl	Island, Grindavík, Blue Lagoon	Wojciech Witaliński
NO.386	*Diaforobiotus* sp.	78°44′02′′ N 16°36′12′′ E 47 m asl	Norway, Svalbard, Ragnardalen	Michala Bryndová
IT.338	*Murrayon* cf. *pullari*	44°23′54.26′′ N 10° 0′23.08′′ E 1594 m asl	Italy, Parma, Corniglio	Matteo Vecchi & Claudio Ferrari

^a^Neotype population.

^b^Candidate neotype population.

### DNA sequencing

Genomic DNA was extracted from individual animals following a Chelex 100 resin (BioRad) extraction method by Casquet et al.^50^ with modifications described in detail in Stec et al.^51^. Each specimen was mounted in water on a temporary microscope slide and examined under light microscope prior to DNA extraction. We sequenced four DNA fragments, three nuclear (18S rRNA, 28S rRNA, ITS-2) and one mitochondrial (COI) from 2–4 individuals per each of the six newly analysed populations. All fragments were amplified and sequenced according to the protocols described in Stec et al.^51^; primers with their original references are listed in Table 4. Sequencing products were read with the ABI 3130xl sequencer at the Molecular Ecology Lab, Institute of Environmental Sciences of the Jagiellonian University, Kraków, Poland. Sequences were processed in BioEdit ver. 7.2.5^52^ and submitted to NCBI GenBank.

**Table 4:** Primers with their original references used for amplification of the four DNA fragments sequenced in the study. COI sequences for all population were amplified with primer set LCO1490-JJ + HCO2198-JJ except *Adorybiotus* population (JP.008) for which LCO1490 + HCOoutout set was used.

DNA marker	Primer name	Primer direction	Primer sequence (5′–3′)	Primer source
18S rRNA	18S_Tar_Ff1	Forward	AGGCGAAACCGCGAATGGCTC	^77^
	18S_Tar_Rr1	Reverse	GCCGCAGGCTCCACTCCTGG	
28S rRNA	28S_Eutar_F	Forward	ACCCGCTGAACTTAAGCATAT	^78,79^
	28SR0990	Reverse	CCTTGGTCCGTGTTTCAAGAC	
ITS-2	ITS2_Eutar_Ff	Forward	CGTAACGTGAATTGCAGGAC	^25^
	ITS2_Eutar_Rr	Reverse	TCCTCCGCTTATTGATATGC	
COI	LCO1490-JJ	Forward	CHACWAAYCATAAAGATATYGG	^80^
	HCO2198-JJ	Reverse	AWACTTCVGGRTGVCCAAARAATCA	
	LCO1490	Forward	GGTCAACAAATCATAAAGATATTGG	^81^
	HCOoutout	Reverse	GTAAATATATGRTGDGCTC	^82^

### Phylogenetic analysis

The phylogenetic analyses were conducted using concatenated 18S rRNA + 28S rRNA + ITS-2 + COI sequences for Macrobiotoidea with *Bertolanius volubilis* (Durante Pasa & Maucci, 1975)^53^, *Eohypsibius nadjae* Kristensen, 1982^54^, *Hypsibius exemplaris* Gąsiorek et al. 2018^28^ and *Ramazzottius subanomalus* (Biserov, 1985)^55^ as outgroups. We choose isolates from the families Richtersiidae and Murrayidae with all four sequenced markers (18S rRNA, 28S rRNA, ITS-2 and COI) and isolates that overlap with the sequences produced in this study in the cases of 18S rRNA and 28S rRNA. However, there were four exceptions to this: two *Adorybiotus granulatus* (Richters, 1903)^32^ isolates, with only 18S rRNA sequences (HQ604961 and HQ604962), that were included as they are the only available sequences for the nominal species of the genus *Adorybiotus* (although the species identification is uncertain; see the Discussion for details). The other two exceptions were *Murrayon dianae* (Kristensen, 1982)^54^ and *Dactylobiotus ovimutans* Kihm et al., 2020^56^ that were included to have a better representation of the Murrayidae. For the family Macrobiotidae, one to two species of each genus, for which sequences are available in GenBank, were included in the analysis (see Table 5 for GenBank accession numbers). In the analysed dataset, 82% (31/38) terminals had sequences for all four markers.

**Table 5:** GenBank accession numbers of the DNA sequences used for phylogeny reconstruction.

Species	18S rRNA	28S rRNA	COI	ITS-2	Source
*Hypsibius exemplaris*^a^	MG800327	MG800337	MG818724	MG800336	^28^
*Ramazzottius subanomalus*	MF001997	MF001998	MF001999	MG432819	^77^
*Bertolanius volubilis*	HQ604918	–	AY598769	–	^6,22^
*Eohypsibius nadjae*	HQ604921	–	–	–	^6^
*Minibiotus ioculator*^a^	MT023998	MT024041	MT023412	MT024000	^51^
*Minibiotus pentannulatus*^a^	MT023999	MT024042	MT023413	MT024001	^51^
*Tenuibiotus voronkovi*	KX810045	KX810049	KX810042	KX810046	^83^
*Tenuibiotus zandrae*^a^	MN443040	MN443035	MN444827	MN443038	^84^
*Paramacrobiotus areolatus*^a^	MH664931	MH664948	MH675998	MH666080	^30^
*Paramacrobiotus fairbanksi*	MH664941	MH664950	MH676011	MH666090	^30^
*Macrobiotus shonaicus*^a^	MG757132	MG757133	MG757136	MG757134	^85^
*Macrobiotus caelestis*^a^	MK737073	MK737071	MK737922	MK737072	^86^
*Xerobiotus pseudohufelandi*	HQ604989	–	AY598776	–	^6,22^
*Mesobiotus harmsworthi*^a^	MH197146	MH197264	MH195150	MH197154	^87^
*Mesobiotus dilimanensis*^a^	MN257048	MN257049	MN257047	MN257050	^88^
*Richtersius coronifer* NO.385^a^	MH681760	MH681757	MH676053	MH681763	^31^
*Richtersius* aff. *coronifer* GR.008	MK211386	MK211384	MK214323–4	MK211380–1	^31^
*Richtersius* aff. *coronifer* IT.120	MH681761	MH681758	MH676054	MH681764	^31^
*Richtersius* aff. *coronifer* IT.317	MK211387	MK211385	MK214326–7	MK211382–3	^31^
*Richtersius* aff. *coronifer* PL.247	MH681762	MH681759	MH676055	MH681765	^31^
*Diaforobiotus islandicus* IS.042^b^	**MT812470**	**MT812461**	**MT808072**	**MT812597**	**This study**
*Diaforobiotus* sp. NO.386	**MT812471**	**MT812463**	**MT808074**	**MT812598**	**This study**
*Diaforobiotus* sp. ID.517	**MT812472**	**MT812462**	**MT808073**	**MT812599**	**This study**
*Murrayon dianae*	FJ435737	FJ435762	FJ435801	–	^89^
*Murrayon* cf *pullari* IT.338	**MT812477**	**MT812465**	**MT808080**	**MT812603**	**This study**
*Dactylobiotus parthenogeneticus* FR.149	MT373694	MT373700	MT373804	MT374191	^90^
*Dactylobiotus parthenogeneticus* GB.003	MT373693	MT373699	MT373803	MT374190	^90^
*Dactylobiotus selenicus* FI.073	**MT812476**	**MT812466**	**MT808076**	**MT812602**	**This study**
*Dactylobiotus ovimutans*^a^	MT136805	–	MT132333	–	^56^
*Crenubiotus* sp. GB.108	**MT812473**	**MT812467**	**MT808077-8**	**MT812604-5**	**This study**
*Crenubiotus crenulatus* NO.429^a^	**MT812474**	**MT812463**	**MT808079**	**MT812606**	**This study**
*Adorybiotus granulatus*	HQ604961–2	–	–	–	^6^
*Adorybiotus* cf. *granulatus* JP.008	**MT812475**	**MT812464**	**MT808075**	**MT812600-1**	**This study**

^a^Type or neotype population.

^b^Candidate neotype population.

The 18S rRNA, 28S rRNA and ITS-2 sequences were aligned using MAFFT ver. 7^57,58^ with the G-INS-i method (thread = 4, threadtb = 5, threadit = 0, reorder, adjustdirection, anysymbol, maxiterate = 1000, retree 1, globalpair input). The COI sequences were aligned according to their aminoacid sequences (translated using the invertebrate mitochondrial code) with the MUSCLE algorithm^59^ in MEGA7^60^ with default settings (all gap penalties = 0, max iterations = 8, clustering method = UPGMB, lambda = 24). Alignments were visually inspected and trimmed in MEGA7. Aligned sequences were concatenated with an in-house R script provided by MV. Model selection and phylogenetic reconstructions were done on the CIPRES Science Gateway^61^. Model selection was performed for each alignment partition (6 in total: 18S rRNA, 28S rRNA, ITS-2 and three COI codons) with PartitionFinder2^62^, partitions and models selection process and results are present in Supplementary Data S1. The BI phylogenetic reconstruction was done with MrBayes v3.2.6^63^ without BEAGLE. Four runs with one cold chain and three heated chains were run for 20 million generations with a burning of 2 million generations, sampling a tree every 1000 generations. Posterior distribution sanity was checked with the Tracer v1.7^64^ and with the R package RWTY^65^. MrBayes input file with the input alignment is available as Supplementary Data S2. ML phylogenetic reconstruction was performed with RAxML-HPC Black Box 8.2.12^66^ with 1000 bootstrap replicates and estimation of proportion of invariable sites (f = a, N = 1000, m = GTRCATI). The phylogenetic trees were visualised with FigTree v1.4.4^67^ and the image was edited with Inkscape 0.92.3^68^. The complete output trees from BI and ML analysis Supplementary Data S3.

### Microscopy and imaging

Specimens for light microscopy were mounted on microscope slides in a small drop of Hoyer’s medium and secured with a cover slip, following the protocol by Morek et al.^69^. Slides were examined under an Olympus BX53 light microscope with phase and Nomarski differential interference contrasts (PCM and NCM, respectively; named collectively as light contrast microscopy, LCM), associated with an Olympus DP74 digital camera. In order to obtain clean and extended specimens for SEM, tardigrades were processed according to the protocol by Stec et al.^49^. Specimens were examined under high vacuum in a Versa 3D DualBeam Scanning Electron Microscope (SEM) at the ATOMIN facility of the Jagiellonian University, Kraków, Poland. All figures were assembled in Corel Photo-Paint X6, ver. 16.4.1.1281. For structures that could not be satisfactorily focused in a single LCM photograph, a vertical stack of 2–6 images were taken with an equidistance of ca. 0.2 μm and assembled manually into a single deep-focus image in Corel Photo-Paint.

### Morphometrics and nomenclature

All measurements are given in micrometres (μm). Sample size was adjusted following recommendations by Stec et al.^70^. Structures were measured only if undamaged and their orientation was suitable. Body length was measured from the anterior extremity to the end of the body, excluding the hind legs. The terminology used to describe oral cavity armature and egg shell morphology follows Michalczyk and Kaczmarek^71^ and Kaczmarek and Michalczyk^72^ respectively. Macroplacoid length sequence is given according to Kaczmarek et al.^73^. Buccal tube length and the level of the stylet support insertion point were measured according to Pilato^74^. The *pt* index is the ratio of the length of a given structure to the length of the buccal tube expressed as a percentage^74^. Measurements of buccal tube widths, heights of claws and eggs follow Kaczmarek and Michalczyk^72^. Morphometric data were handled using the “Parachela” ver. 1.7 template available from the Tardigrada Register^75^ and are given in Supplementary Data S4. Tardigrade taxonomy follows Bertolani et al.^6^ with updates from Guidetti et al.^11^, Vecchi et al.^76^ and Morek et al.^21^.

## Supplementary information

Supplementary Information 1.

Supplementary Information 2.

Supplementary Information 3.

Supplementary Information 4.

## Data Availability

All data generated and analysed during this study are included in the article (and its Supplementary Information files). DNA sequences are deposited and available in GenBank.
