# Supplementary material for: Resolving the systematics of Richtersiidae by multilocus phylogeny and an integrative redescription of the nominal species for the genus Crenubiotus (Tardigrada)
Source: Sci Rep. 2020 Nov 10;10:19418. doi: 10.1038/s41598-020-75962-1 (PMC7655870; doi:10.1038/s41598-020-75962-1)
Supplement: Supplementary file 1 — Supplementary Information 1. [file 41598_2020_75962_MOESM1_ESM.pdf]

Supplementary Data S1. Evolutionary models and partitioning scheme retrieved by PartitionFinder2

Stec D., Vecchi M., Maciejowski W., Michalczyk Ł. - Resolving the systematics of Richtersiidae by multilocus phylogeny and integrative redescription of the nominal species for the genus Crenubiotus (Tardigrada)

Model selection for MrBayes

Settings used

```
alignment        : ./infile.phy
branchlengths    : linked
models           : JC, K80, SYM, F81, HKY, GTR, JC+G, K80+G, SYM+G, F81+G, HKY+G, GTR+G, JC+I,
K80+I, SYM+I, F81+I, HKY+I, GTR+I, JC+I+G, K80+I+G, SYM+I+G, F81+I+G, HKY+I+G, GTR+I+G
model_selection  : aicc
search           : all


Best partitioning scheme

Scheme Name       : 203
Scheme lnL        : -25497.593627929688
Scheme AICc       : 51273.7423809
Number of params  : 133
Number of sites   : 2973
Number of subsets : 6

Subset | Best Model | # sites    | subset id                        | Partition names
1      | GTR+I+G    | 1009       | dbb1f009713256a64f462726a798bb1a | 18S
2      | GTR+G      | 819        | 4d391b446f6180648ddfacd1f9af325c | 28S
3      | GTR+I+G    | 210        | 8249a96feca9aeae948e49b3e375cc7b | COIcodon1
4      | GTR+G      | 210        | 55a4029b1a7e672603190dc88575dc0b | COIcodon2
5      | SYM+I+G    | 209        | 6005fc13df64db8a442b1ecb4b22f43b | COIcodon3
6      | GTR+I+G    | 516        | 5dd563034423d305cc8e6e1d4e17cc34 | ITS2


Scheme Description in PartitionFinder format
Scheme_203 = (18S) (28S) (COIcodon1) (COIcodon2) (COIcodon3) (ITS2);
```

MrBayes block for partition definitions

Warning: MrBayes only allows a relatively small collection of models. If any model in your analysis is not one that is included in MrBayes (e.g. by setting nst = 1, 2, or 6 for DNA sequences; or is not in the available list of protein models for MrBayes)then this MrBayes block will just set that model to nst = 6 for DNA, or 'wag' for Protein. Similarly, the only additional parameters that this MrBayes block will include are +I and +G. Other parameters, such as +F and +X, are ignored. If you want to use this MrBayes block for your analysis, please make sure to check it carefully before you use it we've done our best to make it accurate, but there may be errors that remain!

```
begin mrbayes;

	charset Subset1 = 1-1009;
	charset Subset2 = 1010-1828;
```

Supplementary Data S1. Evolutionary models and partitioning scheme retrieved by PartitionFinder2

Stec D., Vecchi M., Maciejowski W., Michalczyk Ł. - Resolving the systematics of Richtersiidae by multilocus phylogeny and integrative redescription of the nominal species for the genus Crenubiotus (Tardigrada)

```
                    charset Subset3 = 1829-2457\3;
                    charset Subset4 = 1830-2457\3;
                    charset Subset5 = 1831-2457\3;
                    charset Subset6 = 2458-2973;

                    partition PartitionFinder = 6:Subset1, Subset2, Subset3, Subset4, Subset5, Subset6;
                    set partition=PartitionFinder;

                    lset applyto=(1) nst=6 rates=invgamma;
                    lset applyto=(2) nst=6 rates=gamma;
                    lset applyto=(3) nst=6 rates=invgamma;
                    lset applyto=(4) nst=6 rates=gamma;
                    lset applyto=(5) nst=6 rates=invgamma;
prset applyto=(5) statefreqpr=fixed(equal);
                    lset applyto=(6) nst=6 rates=invgamma;

                    prset applyto=(all) ratepr=variable;
                    unlink statefreq=(all) revmat=(all) shape=(all) pinvar=(all) tratio=(all);

end;
```

```
Model selection for RaxML - GAMMA
Settings used

alignment       : ./infile.phy
branchlengths    : linked
models          : JC+G, K80+G, TRNEF+G, K81+G, TVMEF+G, TIMEF+G, SYM+G, F81+G, HKY+G, TRN+G,
K81UF+G, TVM+G, TIM+G, GTR+G
model_selection  : aicc
search          : all


Best partitioning scheme

Scheme Name      : 203
Scheme lnL       : -25542.124877929688
Scheme AICc      : 51349.6775239
Number of params  : 127
Number of sites   : 2973
Number of subsets : 6

Subset | Best Model | # sites    | subset id                    | Partition names
1      | GTR+G      | 1009       | dbb1f009713256a64f462726a798bb1a | 18S
2      | GTR+G      | 819        | 4d391b446f6180648ddfacd1f9af325c | 28S
```

Supplementary Data S1. Evolutionary models and partitioning scheme retrieved by PartitionFinder2

Stec D., Vecchi M., Maciejowski W., Michalczyk Ł. - Resolving the systematics of Richtersiidae by multilocus phylogeny and integrative redescription of the nominal species for the genus Crenubiotus (Tardigrada)

```
3     | TVM+G     | 210       | 8249a96feca9aeae948e49b3e375cc7b | COIcodon1
4     | TVM+G     | 210       | 55a4029b1a7e672603190dc88575dc0b | COIcodon2
5     | SYM+G     | 209       | 6005fc13df64db8a442b1ecb4b22f43b | COIcodon3
6     | GTR+G     | 516       | 5dd563034423d305cc8e6e1d4e17cc34 | ITS2


Scheme Description in PartitionFinder format
Scheme_203 = (18S) (28S) (COIcodon1) (COIcodon2) (COIcodon3) (ITS2);


RaxML-style partition definitions
Warning: RAxML allows for only a single model of rate heterogeneity in partitioned analyses. I.e. all partitions must be assigned one of three types of model: No heterogeneity (e.g. GTR); +G (e.g. GTR+G); or +I+G (e.g. GTR+I+G). If the best models for your datasetcontain different types of model for different subsets you will need to decide on the best rate heterogeneity model before you run RAxML. If you prefer to do things more rigorously, you can run separate PartitionFinder analyses for each type of rate heterogenetity Then choose the scheme with the lowest AIC/AICc/BIC score. Note that these re-runs will be quick!

DNA, Subset1 = 1-1009
DNA, Subset2 = 1010-1828
DNA, Subset3 = 1829-2457\3
DNA, Subset4 = 1830-2457\3
DNA, Subset5 = 1831-2457\3
DNA, Subset6 = 2458-2973
```

```
Model selection for RaxML – GAMMA + I

Settings used

alignment        : ./infile.phy
branchlengths    : linked
models           : JC+I+G, K80+I+G, TRNEF+I+G, K81+I+G, TVMEF+I+G, TIMEF+I+G, SYM+I+G, F81+I+G, HKY+I+G, TRN+I+G, K81UF+I+G, TVM+I+G, TIM+I+G, GTR+I+G
model_selection  : aicc
search           : all


Best partitioning scheme

Scheme Name      : 203
Scheme lnL       : -25496.86407470703
Scheme AICc      : 51272.2832745
```

Supplementary Data S1. Evolutionary models and partitioning scheme retrieved by PartitionFinder2

Stec D., Vecchi M., Maciejowski W., Michalczyk Ł. - Resolving the systematics of Richtersiidae by multilocus phylogeny and integrative redescription of the nominal species for the genus Crenubiotus (Tardigrada)

```
Number of params  : 133
Number of sites   : 2973
Number of subsets : 6

Subset | Best Model | # sites    | subset id                        | Partition names
1      | GTR+I+G    | 1009       | dbb1f009713256a64f462726a798bb1a | 18S
2      | GTR+I+G    | 819        | 4d391b446f6180648ddfacd1f9af325c | 28S
3      | TVM+I+G    | 210        | 8249a96feca9aeae948e49b3e375cc7b | COIcodon1
4      | TVM+I+G    | 210        | 55a4029b1a7e672603190dc88575dc0b | COIcodon2
5      | SYM+I+G    | 209        | 6005fc13df64db8a442b1ecb4b22f43b | COIcodon3
6      | GTR+I+G    | 516        | 5dd563034423d305cc8e6e1d4e17cc34 | ITS2


Scheme Description in PartitionFinder format
Scheme_203 = (18S) (28S) (COIcodon1) (COIcodon2) (COIcodon3) (ITS2);

RaxML-style partition definitions
Warning: RAxML allows for only a single model of rate heterogeneity in partitioned analyses. I.e. all partitions must be assigned one of three types of model: No heterogeneity (e.g. GTR); +G (e.g. GTR+G); or +I+G (e.g. GTR+I+G). If the best models for your datasetcontain different types of model for different subsets you will need to decide on the best rate heterogeneity model before you run RAxML. If you prefer to do things more rigorously, you can run separate PartitionFinder analyses for each type of rate heterogenetity Then choose the scheme with the lowest AIC/AICc/BIC score. Note that these re-runs will be quick!

DNA, Subset1 = 1-1009
DNA, Subset2 = 1010-1828
DNA, Subset3 = 1829-2457\3
DNA, Subset4 = 1830-2457\3
DNA, Subset5 = 1831-2457\3
DNA, Subset6 = 2458-2973
```

```
RaxML model selection:

Gamma
Scheme AICc      : 51349.6775239

Gamma + I
Scheme AICc      : 51272.2832745

The Gamma+I model had a lower AICc so it was chosed for the RaxML analysis
```
